# Commentary: Comparison of conventional ICSI and rescue ICSI in patients without severe male factor and poor oocyte yield

**DOI:** 10.3389/fendo.2025.1719972

**Published:** 2025-11-21

**Authors:** Bangbei Wan, Weiying Lu

**Affiliations:** 1 Reproductive Medical Center, Hainan Women and Children’s Medical Center, Haikou, China; 2 Department of Urology, Haikou Affiliated Hospital of Central South University Xiangya School of Medicine, Haikou, China

**Keywords:** intracytoplasmic sperm injection, fertilization failure, oocytes, male infertility, rescue intracytoplasmic sperm injection

## Introduction

Li et al. retrospectively compared conventional intracytoplasmic sperm injection (ICSI) with early rescue ICSI (R-ICSI, performed ~5–6 h after insemination when no second polar body was observed) in first-cycle patients with poor oocyte yield (3–5 oocytes) and no severe male factor. Across 604 conventional ICSI and 116 early R-ICSI cycles, oocyte maturation (MII), normal fertilization (2PN), embryo morphokinetics, blastocyst formation, and clinical outcomes (pregnancy, ongoing pregnancy, live birth) were broadly similar, whereas multipronuclear (MPN) fertilization was higher after early R-ICSI (6.33% vs. 1.02%) (1). The authors conclude that early R-ICSI achieves comparable reproductive outcomes to conventional ICSI in this population, despite a higher MPN rate.

## Study design, clinical context, and interpretability

### Strengths

The study addresses a frequent, pragmatic dilemma—how to minimize total fertilization failure (TFF) and cycle cancellation when oocyte numbers are limited. Inclusion criteria (first ART cycle; 3–5 oocytes; normal/mildly impaired semen) and clearly defined laboratory protocols (short co-incubation, early PB check at 5–6 h, direct-penetration ICSI for rescue) enhance reproducibility and clinical relevance (1).

### Context

Evidence from randomized and systematic reviews indicates that routine ICSI in non-severe male factor does not improve live birth compared to conventional IVF, supporting a conservative starting strategy that preserves physiologic selection and reserves ICSI for salvage (2, 3). In parallel, large Frontiers data show that low oocyte yield is a predictor of fertilization disorders/TFF, reinforcing the appeal of short-term IVF with contingency early R-ICSI (4).

### Interpretability

A key nuance is confounding by indication: the early R-ICSI cohort first underwent IVF and, by definition, had absent PB extrusion at 5–6 h, whereas the comparison cohort had upfront ICSI. Although baseline demographics were similar, treatment pathways differed at the gamete interface. Multivariable adjustment (e.g., for female age, oocyte retrieval rate, semen kinematics) and mixed-effects modeling at the oocyte level would further stabilize effect estimates beyond p-values and per-cycle percentages (1).

### Timing of rescue, MPN risk, and lab workflow

The authors’ protocol aligns with the emerging consensus that earlier intervention mitigates oocyte aging that undermines late rescue attempts. Early cumulus removal coupled with early R-ICSI at ~5–6 h has been reported as clinically safe, though polyploidy/MPN can be higher—consistent with Li et al.’s findings (5). We note that this 4–6 h interval represents an “early rescue” approach aimed at preventing oocyte degeneration rather than the conventional fertilization assessment performed much later after insemination. This timing difference reflects a pragmatic balance between minimizing oocyte aging and avoiding premature intervention.

In Li et al.’s study, sperm samples used for rescue ICSI were freshly prepared at the time of injection rather than taken from extended incubation. This minimizes the potential accumulation of reactive oxygen species (ROS) and reduces the risk of sperm DNA damage. Prolonged sperm incubation is known to increase oxidative stress and could compromise embryo quality; thus, immediate use is advisable for maintaining gamete integrity.

Given the relatively high incidence of MPN or abnormal pronuclear formation observed after a second injection, potential genetic or chromosomal instability should be acknowledged. Embryos with normal morphology may still harbor genetic or epigenetic abnormalities, and future studies integrating PGT-A or methylation analyses would be valuable to verify the long-term safety and developmental competence of early rescue ICSI.

Pragmatically, centers pursuing this strategy should standardize (i) PB-check timing and criteria, (ii) documentation of injection timing relative to retrieval/insemination, and (iii) counseling on the trade-off between preventing TFF and a modestly higher MPN rate requiring embryo deselection (1).

Where resources allow, split insemination (IVF + ICSI on sibling oocytes) remains a safeguard against TFF while limiting unnecessary ICSI—though it is often impractical with only 3–5 oocytes (6). Decision algorithms that trigger early R-ICSI based on short-incubation IVF observations may strike a workable balance in low-yield cycles (1).

Additionally, in cases where the extrusion of the second polar body is unclear, visualization signs such as the HALO sign may assist embryologists in confirming fertilization events, improving the precision of early assessment.

### Outcomes, reporting, and generalizability

Li et al. report no significant differences in D3 quality, blastocyst formation, or clinical endpoints between groups overall, and broadly similar results across subgroups stratified by oocyte retrieval rate. The higher MPN after early R-ICSI did not translate into worse downstream outcomes. These are reassuring real-world signals (1).

For wider adoption and meta-analytic value, we encourage the authors to:

1. present effect sizes with 95% CIs for all primary and secondary endpoints.
2. provide adjusted comparisons (e.g., logistic/Poisson models) and sensitivity analyses for the subgroup with below-anticipated oocyte retrieval.
3. detail operator experience and lab parameters (pipette geometry, injection modality), as embryologist proficiency can influence MPN and survival; and
4. consider external validation against centers using different insemination timings or early-rescue triggers.

Generalizability is supported by concordance with multicenter RCT and Cochrane conclusions that routine ICSI offers no advantage in non-severe male factor, making a “IVF-first, rescue-if-needed” policy sensible for many units (2). Still, inter-center differences in insemination duration, cumulus removal, and PB-assessment protocols warrant caution when extrapolating absolute rates.

## Discussion

Li et al. provide timely evidence that, in first-cycle patients with limited oocyte yield and no severe male factor, early R-ICSI can safeguard against TFF without compromising embryo development or clinical outcomes, albeit with a higher MPN rate necessitating careful embryo selection. Their results support a pragmatic workflow: begin with short-incubation IVF, audit for PB extrusion at 5–6 h, and deploy R-ICSI for oocytes lacking clear PB release—thereby reserving invasive micromanipulation for those who need it, consistent with modern stewardship of ICSI (1).

Future prospective studies could randomize at the protocol level (e.g., IVF-first with predefined early-rescue criteria vs. upfront ICSI) or incorporate genetic or epigenetic assessments to better define the developmental safety of early rescue ICSI. In the meantime, clinics can integrate Li et al.’s findings into counseling: explain that IVF-first aligns with best evidence in non-severe male factor, that early R-ICSI is an effective safety net, and that a small increase in MPN is expected and managed by standard embryo deselection (2).
